# The Association of Restrained Eating and Overeating during COVID-19: A Cross-Lagged Model

**DOI:** 10.3390/nu13124535

**Published:** 2021-12-17

**Authors:** Yicen Cui, Xinyuan Liu, Guangcan Xiang, Qingqing Li, Mingyue Xiao, Hong Chen

**Affiliations:** 1 Key Laboratory of Cognition and Personality, Ministry of Education, Faculty of Psychology, Southwest University, Chongqing 400715, China; Yicenc97@163.com (Y.C.); liuxiny2019@163.com (X.L.); xgcpsycho@163.com (G.X.); 17784729185@163.com (Q.L.); xiaomy099@163.com (M.X.); 2 School of Psychology, Southwest University, Chongqing 400715, China

**Keywords:** restrained eating, overeating, COVID-19, mortality threat, negative affect

## Abstract

Widespread overeating has been found during the 2019 coronavirus (COVID-19) pandemic. The present study investigated whether pre-pandemic restrained eating (RE) predicted overeating during the pandemic, and further explored the behavioral (mortality threat, negative affect) mechanisms underlying this association. An eight-month longitudinal survey was conducted with a large sample of 616 undergraduates from Southwest university. From September 2019 to April 2020, three measurements were conducted. RE was tested before the pandemic (T1), and data of mortality threat, negative affect, and overeating were collected at the middle (T2) and end of (T3) the COVID-19 crisis in China. The correlation results showed that baseline RE was positively associated with mortality threat, negative affect, and overeating at T2 and T3. Moreover, negative affect and mortality threat were positively correlated with overeating. Results from longitudinal mediation showed that baseline RE would positively predict T3 overeating through T2 negative affect, but not T2 mortality threat. This study supports and extends the counterregulatory eating hypothesis that RE positively predicts future overeating, especially through negative emotions. These findings further reveal the core psychological mechanism underlying this positive RE-overeating relation in the context of COVID-19, indicating that the individuals with higher RE could not cope with negative affect adequately, contributing to more overeating.

## 1. Introduction

The 2019 coronavirus (COVID-19) was initially detected in Wuhan, Hubei Province, China, and spread quickly and widely to other cities, causing many deaths. On 31 January 2020, the World Health Organization declared COVID-19 as a Public Health Emergency of International Concern. In order to reduce the spread of the pandemic, the Chinese government took many preventative measures, such as lockdowns. In April 2020, the lifting of lockdown measures in Wuhan city meant that people were gradually returning to their normal life, indicating that the domestic pandemic situation was under control in China. COVID-19 has brought a great negative impact on people’s daily lives [1]. In terms of eating behaviors, Rodgers et al. (2020) have proposed that the pandemic increased the risk for disordered eating (such as binge eating), disinhibited eating, and emotional eating [2]. Both eating disorders and normal individuals reported abnormal eating behaviors, i.e., increased restricting and binge eating during the pandemic [3]. For example, Robinson et al. (2021) reported negative changes on most participants, and the poor mental health caused by the pandemic predicted more overeating during the lockdown [4]. An investigation in Poland also found almost half of the respondents showed more eating and snacking during the outbreak of COVID-19, with a higher frequency in individuals with excess weight [5]. This might be because regular eating routines might be disrupted due to the restriction of activities and movements, which contributed to unhealthy eating habits [6,7]. Additionally, people might eat more to relieve perceptions of increased fear of contagion, anxiety, and other negative emotions, increasing the likelihood of overeating [8,9]. A COVID-19 Italian online survey also showed that nearly half of the respondents experienced depression, anxiety, or even hypochondria, and consumed more comfort foods to feel better [10].

Restrained eating (RE) refers to the cognitive effort exerted by an individual to eat less than they would like [11]. Although some studies have found a negative correlation between dietary restriction and food intake [12,13], other studies have identified RE as a risk factor for overeating [14,15]. The dual pathways model posits that higher dietary restraint increases the individual’s perceived deprivation, which may lead to counterregulatory eating where individuals violate dieting goals and consume food in a disinhibited state [16]. Evidence from empirical research supported this opinion and suggested that dieting would predict future binge eating or bulimic symptoms [14,15,17]. Andrés and Saldaña (2014) also reported that the individuals who dieted last year were 2.01 times more likely to binge eat than non-dieters [18]. However, a longitudinal study found that dietary restraint did not predict future increases in binge eating [19]. Based on the above inconsistent conclusions on the relationship between RE and overeating, this study examines whether and how RE predicts future overeating in the context of the pandemic.

Stress is increasingly considered as a trigger for overeating [20]. Accumulating research has revealed a strong link between stress exposure and binge eating (for a review, see [21]). The psychological stress faced during the pandemic was mainly derived from the fear of death, that is, mortality-threat-related stress [22]. From the perspective of the terror management health model, individuals would reduce the perceived vulnerability to health threat by displaying a health-oriented response [23]. Supporting this model, Goldenberg, Arndt, Hart, & Brown (2005) showed that reminders of mortality information would restrict women to consume snack food [24]. Conversely, some studies found that mortality threat would lead to people engaging in more unhealthy food intake to mitigate the psychological threats, which acted as a compensatory response [25]. Also, the research on life-history strategy explained that people under mortality-threat stress were likely to adopt a quick life-history strategy where people would show impulsive behaviors and pursue immediate rewards [26,27], such as disinhibited eating [28].

Previous theories have posited that overeating served as a strategy to relieve uncomfortable negative emotions [29,30]. Accumulating research has found a strong link between negative emotions or emotion regulation difficulties and binge eating [31,32,33]. Moreover, longitudinal studies have reported that negative affect could positively predict future binge eating or growth in bulimic symptoms [14,15,34]. Therefore, negative affect has been documented as a risk factor for overeating.

Based on the above inconsistent findings, RE does not necessarily mean success in reducing food intake. Instead, most studies have found a positive relation between RE and overeating [14,15,17]. Mortality-threat-related-stress and negative affect were also thought to be contributing factors to overeating. Therefore, this study aims to investigate whether pre-pandemic RE would positively predict overeating during COVID-19, and whether mortality threat and negative affect caused by the pandemic could mediate this relation. Although the relationship between RE and mortality-threat stress or negative emotions has not been discussed in previous studies, research has suggested that RE, stress, and negative emotions drained self-control resources [35,36,37]. Evidence from previous studies has reported that negative emotions may interrupt the cognitive control applied in dietary restraint [38,39]. Limited cognitive resources were mainly employed to control food intake for individuals with higher RE, which indicated that there were not enough cognitive resources to cope with negative emotions or regulate stress. Indeed, it has been found that RE was associated with more emotion regulation difficulties [40]. Thus, individuals with higher RE may perceive increased mortality-threat-related stress and negative emotions due to their inability to remove them.

The present study explores the prediction of pre-pandemic RE on overeating during the COVID-19 crisis and the behavioral mechanisms (mortality threat and negative affect) underlying this prediction by conducting an eight-month longitudinal investigation. Specifically, three follow-up surveys were conducted at three time points: pre-pandemic (September 2019, T1), at the middle of the COVID-19 crisis (February 2020, T2), and at the end of the COVID-19 crisis (April 2020, T3). Three hypotheses were proposed as follows:

**Hypothesis** **1.** *Pre-pandemic RE would be positively correlated with mortality threat, negative affect, and overeating, and that mortality threat and negative affect would be positively associated with overeating.*

**Hypothesis** **2.** *Pre-pandemic RE would positively predict T3 overeating through T2 mortality threat.*

**Hypothesis** **3.** *T2 negative affect would mediate the prediction of pre-pandemic RE on T3 overeating.*

## 2. Method

### 2.1. Participants

The data set was from an ongoing Chinese personality Behavioral-Brain Research Project from Southwest University, Chongqing, China. The entire data collection consisted of three phases. Initially, 901 undergraduates participated in the first collection before the pandemic (from September to December 2019; T1) when the demographic characteristics (e.g., age, sex, and BMI) and RE were measured. Then, 751 of them took the second online behavioral test (i.e., mortality threat, negative affect, and overeating) at the middle of the pandemic crisis (February 2020; T2). Finally, 620 subjects accepted the same test (i.e., mortality threat, negative affect, and overeating) at the end of the pandemic crisis (April 2020; T3). Four subjects were excluded because of missing values in the Restraint Scale, and the remaining 616 subjects were included in the formal analysis. All participants reported no history of neurological or psychiatric disorders. They signed an informed consent document before participating in the experiments and were paid at the end of data collection. This study complied with the standards for the ethical treatment of human participants and was approved by the Ethical Committee of Scientific Research at Southwest University (Project identification code: SWUPSY19090201).

### 2.2. Behavior Measurements

Demographic characteristics for age and sex were collected via self-reports. Body mass index (BMI) was measured with a medical body composition analyzer (M515; seca, Hamburg, Germany).

RE was evaluated with a revised 10-item Restraint Scale [41]. Participants were asked to rate their eating behavior based on their current status, with higher scores reflecting more RE. The Cronbach’s alpha for the current sample was 0.75.

Overeating was measured with one subscale (i.e., uncontrolled eating) of the Chinese version of the TFEQ-R18 in this study (e.g., “Sometimes once I start eating, I can’t stop”). All participants were asked to rate nine items on a four-point Likert scale. The Cronbach’s alpha in this study was 0.916 and 0.92 for uncontrolled eating at T2 and T3, respectively.

The Positive and Negative Affect Scale—Chinese [42] was used to measure participants’ recent negative emotional states. The participants were presented with 10 items measuring negative affect and rated their recent state from 1 (never) to 5 (always). In the present study, Cronbach’s alpha for negative affect was 0.915 and 0.930 at T2 and T3, respectively.

Mortality threat was tested with four questions (e.g., “To what extent do you feel the environment has become more dangerous?”), which derived from Wang and Chen (2016) [43]. All four items were rated on a seven-point Likert scale ranging from 1 (no feel) to 7 (very strong). This scale has reported high reliability in a study regarding Chinese COVID-19 [44]. In this study, the Cronbach’s alpha for mortality threat was 0.924 and 0.941 at T2 and T3, respectively.

### 2.3. Data Analyses

The descriptive statistics and correlation analyses were conducted using SPSS 22.0. The longitudinal cross-lagged path analysis was performed to examine the hypotheses with M-plus 7.0. Pre-pandemic RE (T1), mortality threat, negative affect, and overeating (T2 and T3) were included in the analysis, controlling for age, sex, and BMI. Initially, we calculated the cross-sectional correlations between the study variables at the same time point. Then, the construct stability for mortality threat, negative affect, and overeating from T2 to T3 (i.e., auto-regressive path analysis) and structural panels for these variables (i.e., cross-lagged path analysis) were also examined. Model fit was evaluated by the chi-square, comparative fit index (CFI), the Tucker-Lewis index (TLI), root–mean–square error of approximation (RMSEA), and standardized root–mean–square residual (SRMR). According to the standards proposed by Hu and Bentler (1999), CFI and TLI ≥ 0.95, RMSEA ≤ 0.10, and SRMR ≤ 0.10 indicate a good fit [45]. Then, we constructed a longitudinal mediation model to assess whether the prediction of RE on T3 overeating could be mediated by T2 mortality threat and negative affect. RE was considered as a predictor, T3 overeating as a dependent variable, and mortality threat and negative affect at T2 as mediators. The bootstrapping analysis with 5000 iterations was adopted to calculate the indirect effect and the bias was corrected at a 95% confidence interval (CI). If the CI of the indirect effect did not include zero, the mediating effect was significant.

## 3. Results

The descriptive statistics and correlation coefficients for behavioral variables are reported in Table 1. Results from the correlation analysis showed that baseline RE was positively associated with mortality threat, negative affect, and overeating at T2 and T3. Both mortality threat and negative affect were positively correlated with overeating. Moreover, mortality threat was positively related to negative affect.

**Figure 1:**
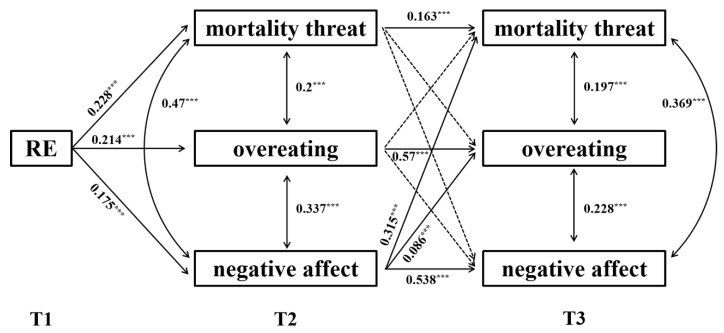
The cross-lagged model of the RE-overeating association. The figures represent the standardized regression coefficients of significant paths. RE = restrained eating. *** *p* < 0.001. **T1**, pre-pandemic; **T2**, the middle of the COVID-19 crisis; **T3**, the end of the COVID-19 crisis.

The cross-lagged model was generated as displayed in Figure 1. The model fit was acceptable, *χ^2^* (18) = 41.13, *p* = 0.0015, CFI = 0.978, TLI = 0.952, RMSEA = 0.046, SRMR = 0.041. The results showed that the auto-regressive effects for mortality threat, negative affect, and overeating were significant (*p* < 0.001). The cross-lagged path analysis revealed that pre-pandemic RE positively predicted mortality threat (*β* = 0.228, *p* < 0.001), negative affect (*β* = 0.175, *p* < 0.001), and overeating (*β* = 0.214, *p* < 0.001) at T2. The T2 negative affect positively predicted mortality threat and overeating at T3. Importantly, pre-pandemic RE predicted T3 overeating through T2 negative affect (indirect effect = 0.016; 95% CI = 0.004, 0.034), but not through T2 mortality threat (indirect effect = −0.013; 95% CI = −0.03, 0.001).

## 4. Discussion

The present study investigated whether pre-pandemic RE would positively predict overeating during the COVID-19 crisis with mediation by mortality threat and negative affect. Two important results were found in the present study and were partially consistent with our hypotheses. First, baseline RE was positively associated with mortality threat, negative affect, and overeating at the middle and end of the pandemic crisis (T2 and T3). Moreover, negative affect and mortality threat were positively correlated with overeating. Second, baseline RE positively predicted T3 overeating through T2 negative affect, but not through T2 mortality threat.

Consistent with H1, the current results showed that pre-pandemic RE was positively associated with mortality threat, negative affect, and overeating at T2 and T3. On one hand, our results supported the opinion that RE would lead to counterregulatory eating [16] and concurred with previous longitudinal studies demonstrating that RE could positively predict future overeating [14,15,17]. However, the current results were inconsistent with the findings of Spoor et al. (2006), this might be due to the fact that our study was carried out under specific stressful situations, and the sample size was large. In line with this interpretation, several studies reported that the pandemic exacerbated the onset of overeating [4]. Therefore, the pandemic might magnify this positive relationship between RE and overeating. On the other hand, RE was associated with an inability to regulate negative stress and emotions. According to existing studies, both maintaining dietary goals and regulating negative stress or emotions consumed limited cognitive resources [35], so more cognitive control resources for dietary restriction meant less cognitive resources for regulating negative emotions and stress. Thus, less resources were devoted to dealing with negative emotions resulting in more unresolved negative emotions, which might account for the positive relation of RE and negative state (mortality threat and negative affect) in this study.

Our findings confirmed H3 rather than H2, suggesting that RE positively predicted future overeating through more negative emotions rather than mortality threat. Interestingly, mortality threat was associated with overeating within the same period but was not related to future overeating. Corroborating existing studies [25,46], the individuals that perceived a higher mortality threat exhibited more impulsive behaviors (i.e., overeating). However, mortality threat caused by COVID-19 represented a temporary acute stress [47], which could not affect people’s eating behaviors in a long term. As predicted, negative affect may be the key mechanism mediating the positive relation between RE and overeating. Higher RE was associated with terrible emotion regulation, which meant that more overeating was needed to alleviate unsolved negative emotions. As explained above, the individuals with higher RE would perceive more unsolved negative emotions due to limited cognitive resources. Some reviews have regarded overeating as an effective strategy to down-regulate negative emotions [48,49]. For example, Booth (1994) has proposed a learning theory suggesting that overeating yields a positive regulatory function to counter negative emotions [50]. Furthermore, excessively consuming “comfort food” has been found to reduce emotional stress through increased serum levels of glucocorticoids [51]. Thus, the individuals who perceived higher negative affect during COVID-19 would eat more to eliminate this negative state. In addition, negative affect would interfere with the cognitive control needed for dieting [38]. For example, Smith et al. (2018) found that negative affect would lead to a reduction of dieting intentions [39]. Therefore, it may be inferred that the inability to relieve negative affect is one of the key psychological mechanisms underlying the individuals with higher RE that exhibit more overeating behaviors in the context of COVID-19.

RE requires a great deal of cognitive resources to withhold food intake [52]. If individuals with higher RE directed sufficient cognitive resources to regulate their eating, they would achieve their dieting goals successfully. Conversely, impaired eating regulation might lead to the occurrence of overeating [53]. The boundary model of eating has proposed emotions may impair the regulation of eating [33,48]. In the context of COVID-19, individuals had to consume their limited cognitive resources to cope with the persistent negative emotions generated by the pandemic, which impaired their dietary regulation ability [49,54]. Sproesser, Strohbach, Schupp, & Renner (2011) suggested that both a lack of self-control resources and a higher need for negative emotion regulation contributed to unhealthy eating patterns [55]. Therefore, the exhausted cognitive control resources triggered by regulating negative affect may be the key reason for the positive RE-overeating relation. The goal conflict model of eating suggests that individuals with RE fail to suppress their food intake due to higher hedonic-eating reward motivation and reduced inhibition control [56]. This model is also supported by the negative relation between self-control and disinhibited eating [28]. Consequently, we speculated that individuals with higher RE failed to deal with negative affect adequately due to limited cognitive resources, interrupting dietary restriction maintenance and exhibiting more overeating.

Overall, this is the first study to explore the longitudinal association of RE and overeating in the context of the COVID-19 pandemic and highlights the importance of negative affect for overeating in individuals with RE. However, it is noted that there are several limitations in this study. First, the measurements depended on self-reported online questionnaires. Despite adequate reliability of the measurements based on self-reports, other methods with higher ecological validity should be adopted in future studies. Second, the sample was included only college students, but the outbreak has affected almost everyone in China. Accordingly, it is worthwhile to test whether the current results can be generalized to other samples across different ages and careers. Third, this study did not measure negative affect, mortality threat, overeating at T1, and restrained eating during COVID-19, which might be one of the reasons why the current results were inconsistent with the findings of Spoor et al. (2006). Thus, it is hard to exclude the influence of temporal stability of these factors when exploring the longitudinal association between RE and future overeating in the present study. Consequently, we hope that future research aims to deeply reveal the causal relationship among these factors through follow-up studies. Finally, previous studies have proposed many factors that would lead to overeating for individuals with RE; however, few studies have suggested solutions. Therefore, future research should focus on which factors could moderate or eliminate this negative impact or develop effective interventions to help individuals reduce food intake successfully.

In conclusion, this study conducted an eight-month longitudinal survey to explore the association of RE and future overeating in the context of COVID-19. Ultimately, we found that pre-pandemic RE positively predicted overeating during the pandemic, and highlighted mediation by negative affect, which supports the counterregulatory eating hypothesis. These findings further reveal the core psychological mechanism underlying the positive RE-overeating association in the context of COVID-19. Individuals with higher RE were unable to adequately alleviate negative affect caused by the pandemic, which contributed to unhealthy eating patterns.

## Figures and Tables

**Table 1:** Descriptive statistics and correlation coefficients of the study variables (*N* = 616, female = 431).

	Variables	M (SD)	1	2	3	4	5	6	7	8
1	Age	18.91 (0.9)	-							
2	BMI	21.19 (2.8)	0.115 **	-						
3	RE (T1)	11.75 (5.5)	0.060	0.431 ***	-					
4	NA (T2)	25.41 (8.6)	0.112 **	−0.031	0.175 ***	-				
5	NA (T3)	25.86 (9.2)	0.021	−0.051	0.088 *	0.528 ***	-			
6	MT (T2)	16.23 (5.3)	0.054	−0.009	0.228 ***	0.491 ***	0.216 ***	-		
7	MT (T3)	12.22 (5.5)	−0.008	−0.019	0.103 *	0.400 ***	0.488 ***	0.321 ***	-	
8	Overeating (T2)	19.10 (5.7)	−0.028	0.042	0.214 ***	0.362 ***	0.236 ***	0.239 ***	0.166 ***	-
9	Overeating (T3)	19.23 (5.8)	−0.019	0.082^*^	0.196 ***	0.262 ***	0.319 ***	0.121 **	0.253 ***	0.591 ***

Notes: M = mean; SD = standard deviation. RE = restrained eating, NA = negative affect, MT = mortality threat. * *p* < 0.05; ** *p* < 0.01; *** *p* < 0.001.

## Data Availability

The data that support the findings of this study are available from the corresponding author upon reasonable request.
